# Application of the quality control circle model to reduce non-conforming surgical instrument packaging and hospital-acquired infection risk: a pilot study

**DOI:** 10.3389/fcimb.2026.1775286

**Published:** 2026-02-20

**Authors:** Lidong Mai, Xiangyun Shen, Chu Zeng, Chubin Lin, Xizhe Huang, Chuyun Pan

**Affiliations:** Central Sterile Supply Department, Shenzhen People’s Hospital (The First Affiliated Hospital, Southern University of Science and Technology; The Second Clinical Medical College, Jinan University), Shenzhen, Guangdong, China

**Keywords:** central sterile supply department (CSSD), healthcare-associated infections (HAIS), quality control circle (QCC), sterile packaging quality improvement, surgical site infection prevention

## Abstract

**Background:**

Healthcare-associated infections (HAIs), particularly postoperative infections, remain a major global concern, and deficiencies in surgical instrument packaging represent an important and preventable risk factor. The Central Sterile Supply Department (CSSD) plays a critical role in maintaining sterile assurance; however, process-related packaging non-conformance can compromise patient safety. This pilot study aimed to apply the Quality Control Circle (QCC) model to reduce non-conforming surgical instrument packaging and strengthen infection prevention capability in a tertiary hospital CSSD.

**Methods:**

This prospective quality improvement study was conducted in the CSSD of a tertiary hospital. Baseline performance was assessed using routine quality monitoring data, with non-conforming packaging defined as missing instruments, incorrect instrument type, nonfunctional instruments, improper sealing, wet packs, labeling errors, or chemical indicator defects. QCC methodology was applied, incorporating Pareto analysis, root cause analysis, structured training, workflow optimization, equipment maintenance reinforcement, and strengthened verification systems. The primary outcome was the non-conforming packaging rate before and after intervention.

**Results:**

Baseline analysis identified missing instruments, incorrect instrument types, and incomplete or nonfunctional instruments as dominant contributors to packaging defects, with an overall baseline non-conformance rate of 0.213%. Following QCC implementation, the rate decreased to 0.199% during the initial assessment (May 2024; 19,060 packs inspected, 38 defective) and further declined to 0.106% during extended follow-up (June–October 2024; 82,182 packs inspected, 87 defective), achieving the predefined improvement target of a 50% reduction. Post-intervention Pareto analysis demonstrated a marked decrease in dominant defect categories, accompanied by strengthened process standardization and stability.

**Conclusion:**

Application of the QCC model significantly improved surgical instrument packaging quality, reduced non-conforming events, and enhanced operational reliability in the CSSD. These findings demonstrate that structured, team-based quality management is feasible, sustainable, and potentially beneficial for supporting infection risk reduction and perioperative safety. The model provides a replicable framework for broader implementation and future outcome-linked research.

## Introduction

Healthcare-associated (nosocomial) infections remain a persistent global challenge and represent a major threat to patient safety (Sandu et al., 2025), particularly in postoperative settings where sterile field integrity is essential (Sartelli et al., 2023). Surveillance data indicate that on any given day in the United States approximately 1 in 31 hospitalized patients has at least one healthcare-associated infection (HAI), corresponding to an estimated 687,000 infections and about 72,000 infection-related hospital deaths annually (Cagle et al., 2022; CDC, 2024). International evidence suggests that the global prevalence of HAIs remains around 7–10% among admitted hospital patients and mortality rates from 7% to 64.6% were reported (Al-Tawfiq, 2025), while rates are significantly higher in low- and middle-income countries where infection prevention systems are often under-resourced (Ahmed et al., 2021; Odoom et al., 2025). In China, large multicenter prevalence surveys have reported inpatient HAI prevalence ranging approximately from 1.6% to 3.1%, with substantially higher infection burdens in intensive care and surgical units. Surgical site infections (SSIs) constitute one of the most frequent postoperative HAIs, commonly reported in 2–5% of surgical procedures in developed healthcare systems, while several Chinese studies have documented higher rates, frequently exceeding 4–6% depending on region, procedure type, and infection control performance (Wang et al., 2018; Dong and Liu, 2025; Yao et al., 2025a). These infections prolong hospitalization, increase antimicrobial exposure and healthcare expenditure, and contribute significantly to morbidity and mortality. Within this context, the CSSD plays a decisive role in infection prevention by ensuring the cleaning, disinfection, functional verification, sterilization, and packaging of reusable surgical devices. Surgical instruments function as extensions of the surgeon’s operative capability; therefore, any compromise in sterile barrier integrity directly increases the potential for microbial contamination and postoperative infection (Rutala and Weber, 2016; Shuai et al., 2025). Previous analyses in Chinese hospitals have demonstrated that packaging-related non-conformities including missing instruments, incorrect instrument types, malfunctioning devices, wet packs, improper sealing, and labeling errors are among the most important risk factors affecting surgical device safety (Zhu et al., 2019; Chen et al., 2022). Logistic regression reports indicate that incorrect packaging methods may increase packaging failure risk more than threefold, while incomplete instrument documentation and mismatched instrument specifications also significantly elevate risk. These findings highlight surgical device packaging quality as a measurable, improvable, and clinically meaningful target within hospital infection prevention systems (Chen et al., 2022; Yi et al., 2022). Quality Control Circle (QCC) methodology, originating in industrial quality management and increasingly applied in clinical healthcare, emphasizes frontline participation, structured root-cause analysis, data-driven improvement, and sustainable performance monitoring. Previous healthcare applications of QCC have demonstrated meaningful reductions in process errors and improvements in clinical quality; however, its application in CSSD management and its potential effect on infection risk remain insufficiently reported (Ou et al., 2025). Keepin mind all this factor and figures CSSD of the healthcare system, this pilot study applied the QCC model in the CSSD of a tertiary hospital to reduce non-conforming surgical instrument packaging and evaluate its potential contribution to hospital-acquired infection risk reduction. The study aimed to identify primary causes of packaging non-conformity, implement targeted improvement measures, and assess measurable outcomes following intervention.

## Methods

This study involved system improvement without patient-identifiable data or direct patient intervention. Institutional approval for QCC-based quality improvement activities was obtained in accordance with hospital policy, and ethical risk was considered minimal.

### Study design and setting

This investigation was designed as a prospective quality improvement pilot single center study for cleaning, disinfection, functional inspection, packaging, sterilization, and distribution of reusable surgical devices across all surgical departments in our hospital. The QCC intervention was implemented in accordance with hospital quality management policy and national CSSD guidelines. The activity period covered baseline assessment, intervention implementation, and follow-up tracking between November 11, 2023 and June 10, 2024.

### Formation of the QCC team

A multidisciplinary Quality Control Circle was established, consisting of CSSD well trained nurses, technicians, senior sterilization staff, quality supervisors, and managerial representatives (**Supplementary Table 1**). All members received training in QCC methodology and average age 40 yrs, including problem identification, Pareto analysis, root-cause analysis, goal setting, solution design, implementation monitoring, and continuous improvement. Team activity followed the PDCA (Plan–Do–Check–Act) framework.

### Baseline situation assessment

Baseline data were obtained from routinely documented quality inspection records of the CSSD. Packaging non-conformance was defined as the presence of any deviation affecting the integrity, accuracy, or safety of the sterile surgical device set. Specifically, non-conforming packages included those with missing instruments, incorrect instrument types, incomplete or malfunctioning instruments, improper sealing, wet packs, labeling errors, or chemical indicator card defects. A Pareto analysis was conducted to identify the primary contributors to packaging failure. The results demonstrated that three categories accounted for the majority of non-conforming events, namely missing instruments, incorrect instrument types, and incomplete or nonfunctional instruments, indicating that errors predominantly originated from human operational factors, process inconsistencies, and inadequate verification procedures. The overall baseline non-conforming packaging rate was calculated as 0.213%, based on the formula $\frac{\text{non-conforming packs}}{\text{total inspected packs}}\times 100$, and this value was used as the reference benchmark for subsequent quality improvement evaluation in this study.

### Goal setting and root cause analysis

The improvement objective was established using conventional QCC computational methodology, which integrates the current defect rate, improvement priority coefficient, and circle capability index to derive a realistic and achievable target. Based on these parameters, the calculated target rate for non-conforming surgical instrument packaging was determined to be 0.106%, representing an anticipated 50% reduction from the baseline rate of 0.213%. To guide targeted intervention design, a comprehensive root cause analysis was subsequently undertaken using fishbone diagrams and structured RCA techniques encompassing personnel, process, environment, equipment, management, and system dimensions (**Supplementary Figure 1**). Each identified contributing factor was evaluated using standardized scoring criteria (n=1 minimal relevance, n=3 moderate relevance, and n=5 major relevance), and true root causes were confirmed when the Pareto cumulative contribution reached ≥ 80% and the total score was ≥ 26 points. This analysis identified several predominant determinants of packaging non-conformance, including insufficient systematic staff training, failure to strictly adhere to packaging inspection protocols, imperfect supervisory mechanisms, inadequate device maintenance, and deterioration of identification and labeling systems. These validated causes provided the scientific basis for subsequent quality improvement strategies within the QCC framework.

### Intervention measures

Targeted Quality Control Circle interventions were designed and implemented using the 5W1H framework to ensure systematic, practical, and evidence-based execution. The primary strategies included the development and standardization of updated surgical instrument lists, accompanied by clear labeling reminders to reduce configuration errors. In parallel, double-checking and supervisory mechanisms were strengthened to enhance verification rigor and accountability. To address equipment-related risks, maintenance procedures and protective measures were reinforced, ensuring timely inspection and preservation of device integrity. Workflow optimization was achieved through refinement of handover protocols and process standardization, thereby improving operational consistency and reducing omission risk. Furthermore, structured personnel training programs, competency assessments, and mentoring reinforcement were introduced to improve staff proficiency, adherence to protocols, and sustained quality awareness. All interventions were implemented in phased stages, with continuous compliance audits conducted throughout the process to monitor effectiveness, ensure fidelity to interventions, and support continuous improvement.

### Outcome measures

The primary outcome measure of this study was the rate of non-conforming surgical device packaging, defined as the proportion of defective instrument packs relative to the total number of inspected packs, calculated using the formula: non-conforming packs/total inspected packs×100%. Following implementation of the QCC interventions, quality monitoring demonstrated a progressive decline in packaging defects. During the initial post-intervention evaluation period in May 2024, a total of 19,060 surgical device packs were inspected, of which 38 were identified as non-conforming, corresponding to a defect rate of 0.199%. Continued monitoring between June and October 2024 further confirmed sustained improvement, with 82,182 packs inspected and 87 non-conforming events recorded, yielding a reduced defect rate of 0.106%, thereby achieving the predefined improvement target. Trends in quality performance were additionally verified using repeated Pareto comparative analyses before and after intervention, demonstrating a marked reduction in the dominant categories of packaging non-conformance and confirming the effectiveness and stability of the implemented quality improvement measures.

### Statistical analysis

All data were obtained from CSSD quality monitoring records. Descriptive statistics were applied to compare pre- and post-intervention defect rates. Improvement effectiveness was expressed as percentage reduction relative to baseline. Data integrity was verified by double entry and supervisory review.

## Results

### Baseline performance and dominant defect distribution

**Figure 1:**
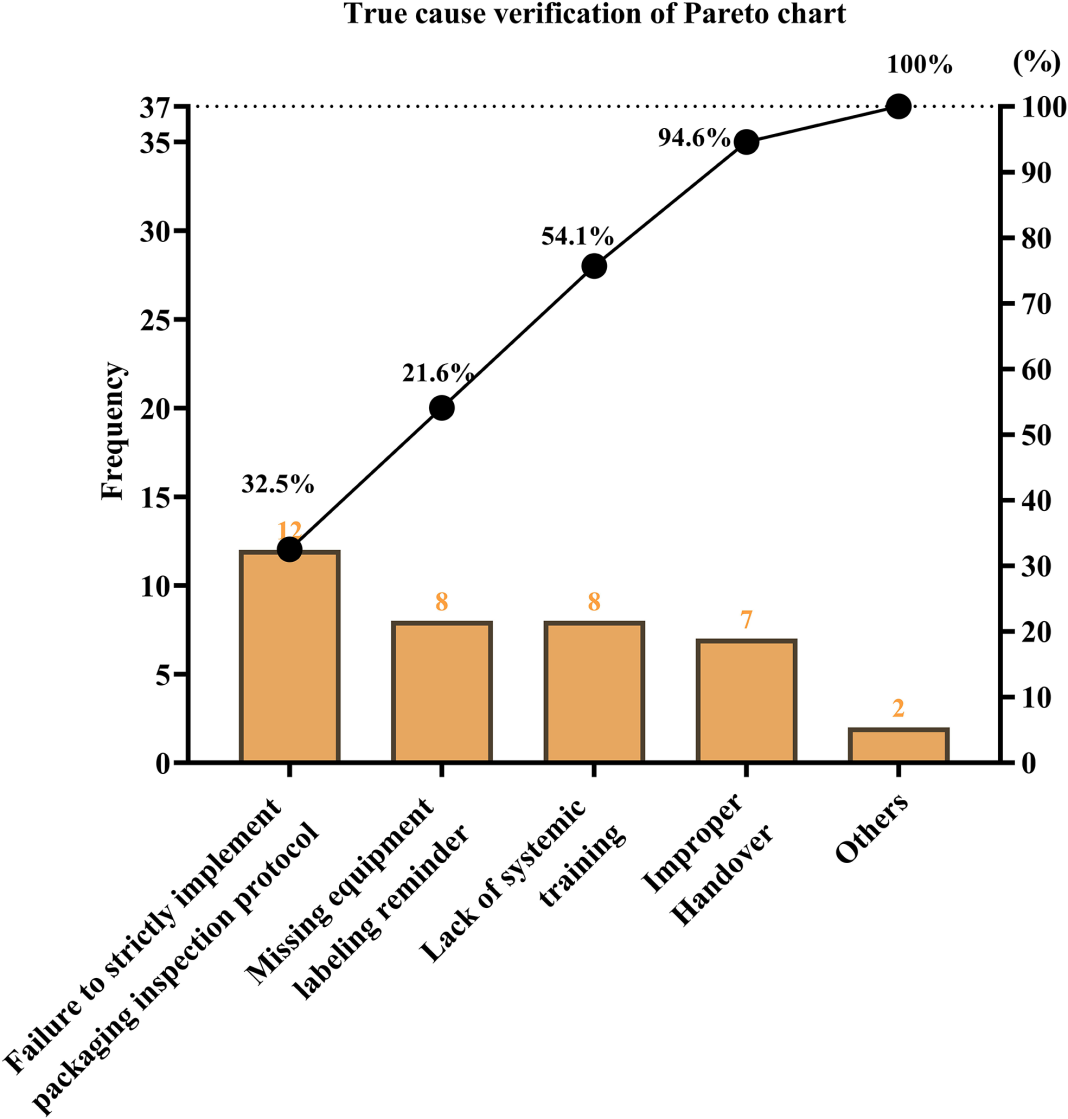
Baseline distribution of packaging defects in CSSD prior to QCC intervention. Pareto analysis showing dominant defect categories including missing instruments, incorrect instrument types, and nonfunctional or incomplete instruments.

Baseline evaluation of CSSD quality monitoring records demonstrated that packaging non-conformance was primarily concentrated in three major categories: missing instruments, incorrect instrument types, and incomplete or nonfunctional instruments. Pareto analysis illustrated that these three defects accounted for the majority of total quality failures, confirming them as key contributors to packaging risk (**Figure 1****;****Table 1**). Additional but less frequent defects included improper sealing of sterilized items, wet packs, labeling errors, and chemical indicator card issues. The overall pre-intervention non-conforming packaging rate was 0.213%, which was used as the baseline reference value for subsequent improvement comparison. The improvement key point value, representing the proportion of defects amenable to intervention, was determined to be 81.9%, while the circle competency, reflecting team capability to execute improvement measures, was 60.6%. Target Value = Current Value − (Current Value× Circle Competency × Improvement Key Points). Substituting actual parameters: 0.213%−(0.213%×60.6%×81.9%) = 0.106%. Thus, the computed target defect rate was 0.106%, corresponding to an anticipated 50% reduction relative to baseline based. Achievement analysis demonstrated that the post-intervention non-conforming rate reached this target level, confirming that the predicted improvement capability accurately reflected actual performance.

**Table 1:** Baseline distribution and frequency of non-conforming surgical instrument packaging prior to intervention.

Subjects	Times	Proportion	Cumulative percentage
Failure to strictly implement packaging inspection protocol	12	32.5%	32.50%
Missing equipment labeling reminder	8	21.6%	54.10%
Lack of systemic training	8	21.6%	75.70%
Improper handover	7	18.9%	94.60%
Others	2	5.4%	100.00%
Sum	37	100.00%	

Data are derived from routine CSSD quality monitoring records.

### Achievement of quantitative improvement objectives

**Figure 2:**
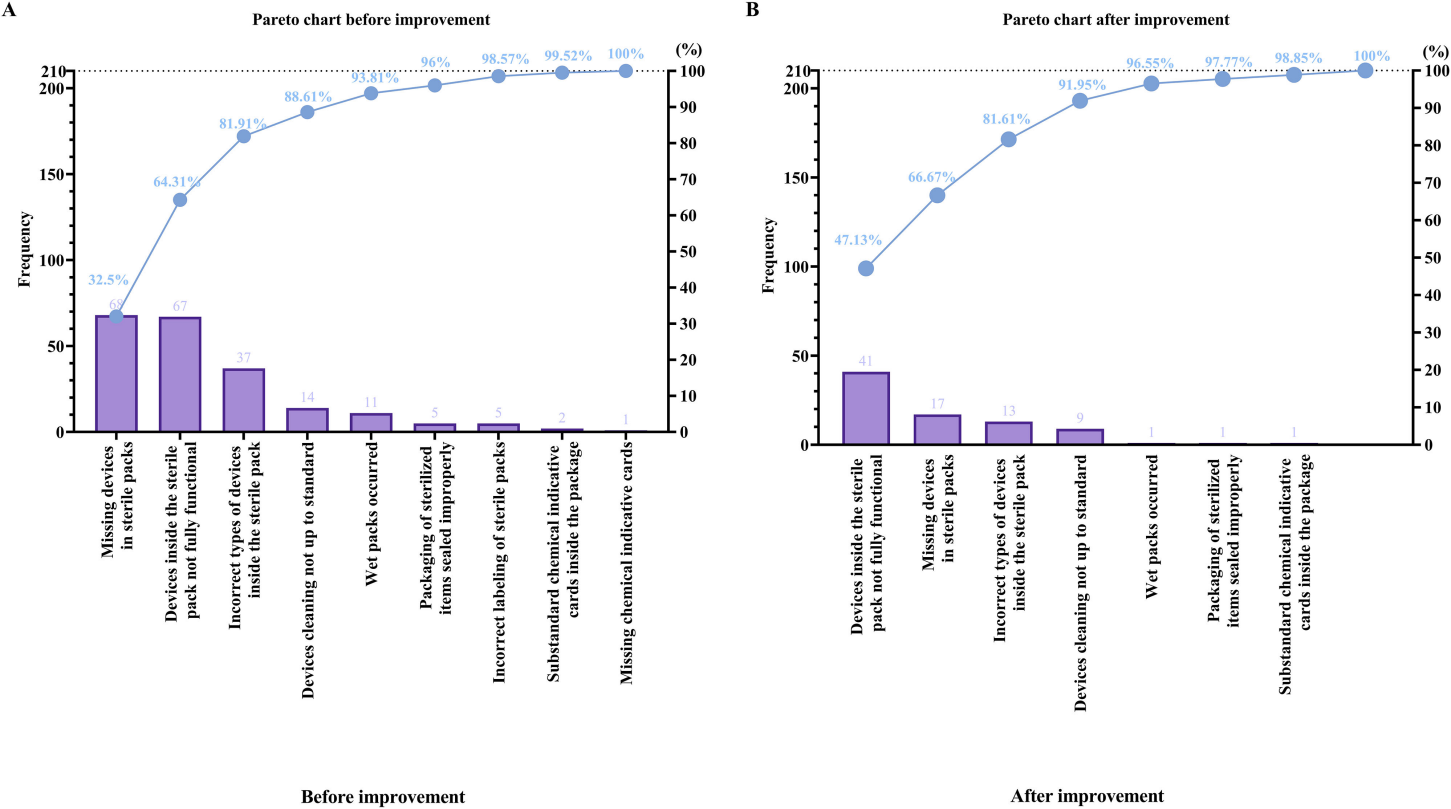
Comparison of packaging defect composition before and after QCC implementation. **(A)** Pareto chart showing pre-intervention defect distribution, with missing devices, non-functional instruments, and incorrect device types constituting the majority of defects. **(B)** Pareto chart showing post-intervention reduction in major defect categories and overall optimization of defect structure.

Following implementation of the QCC interventions, a progressive and sustained reduction in packaging defects was observed. During the first post-rectification evaluation period (May 1–31, 2024), a total of 19,060 surgical device packs were inspected (**Supplementary Table 2**), among which 38 were identified as non-conforming, yielding a defect rate of 0.199%. Among these 38 defects, the most frequent category was “devices inside the sterile pack not fully functional” (11 cases, 28.95%), followed by missing devices (10 cases, 26.31%), incorrect instrument types (5 cases, 13.15%), devices not cleaned to standard (4 cases, 10.52%), wet packs (3 cases, 7.90%), improper sealing of sterilized packs (3 cases, 7.90%), and substandard chemical indicator issues (2 cases, 5.27%). Cumulative percentage analysis confirmed that the first three categories together accounted for 68.41% of all post-rectification defects, consistent with the pre-identified dominant risk areas (**Figure 2**; **Table 2**). Continued continuous monitoring during the extended follow-up period from June to October 2024 demonstrated further improvement and stabilization of quality performance. Across 82,182 inspected packs, only 87 non-conforming events were recorded, corresponding to a markedly reduced defect rate of 0.106%, thereby successfully achieving the predefined 50% reduction target relative to baseline. During this period, nonfunctional or incomplete instruments remained the most frequent issue (41 cases; 47.13%), followed by missing instruments (17 cases; 19.54%), incorrect instrument types (13 cases; 14.94%), and less frequent occurrences of inadequate cleaning (9 cases; 10.34%), improper sealing (4 cases; 4.60%), wet packs (1; 1.15%), substandard chemical indicator cards (1; 1.15%), and missing indicator cards (1; 1.15%). Cumulative contribution analysis showed that the top three categories accounted for over 81% of all defects, indicating targeted control of principal risk components and confirming structural optimization of defect composition (**Figure 2**; **Table 2**). These findings collectively verify that the improvement objective established during the planning stage was both realistic and scientifically achievable, and that the implemented QCC measures produced stable, sustained quality enhancement.

**Table 2:** Tangible quality outcomes and achievement of improvement target following QCC intervention.

Packaging defect category	May 2024 n (%)	June–October 2024 n (%)
Devices inside the sterile pack not fully functional	11 (28.95%)	41 (47.13%)
Missing devices in sterile packs	10 (26.31%)	17 (19.54%)
Incorrect types of devices inside the sterile pack	5 (13.15%)	13 (14.94%)
Devices cleaning not up to standard	4 (10.52%)	9 (10.34%)
Wet packs occurred	3 (7.90%)	1 (1.15%)
Packaging of sterilized items sealed improperly	3 (7.90%)	4 (4.60%)
Substandard/missing chemical indicative cards	2 (5.27%)	2 (2.30%)
Total defects	38 (100%)	87 (100%)
Defect rate (%)	0.199% (38/19,060)	0.106% (87/82,182)

### Structural optimization of defect composition

Beyond reducing the overall defect rate, a substantial optimization in the composition of quality failures was achieved. Before intervention, Pareto analysis showed that the three dominant high-risk categories nonfunctional or incomplete devices, missing instruments, and incorrect instrument types occurred 68, 67, and 37 times, respectively, together contributing 81.91% of all defects. After implementation of the QCC strategy, these categories declined to 41, 17, and 13 cases, representing 47.13%, 19.54%, and 14.94% of post-intervention events, with the cumulative contribution controlled at 81.61%. Meanwhile, lower-frequency defects such as inadequate cleaning, improper sealing, wet packs, and chemical indicator problems were also markedly suppressed. The post-intervention Pareto curve demonstrated a visibly flatter trend compared with baseline, confirming strengthened packaging accuracy, improved verification compliance, and enhanced reliability of instrument functionality (**Figure 2****).**

### Process standardization and quality stability

**Figure 3:**
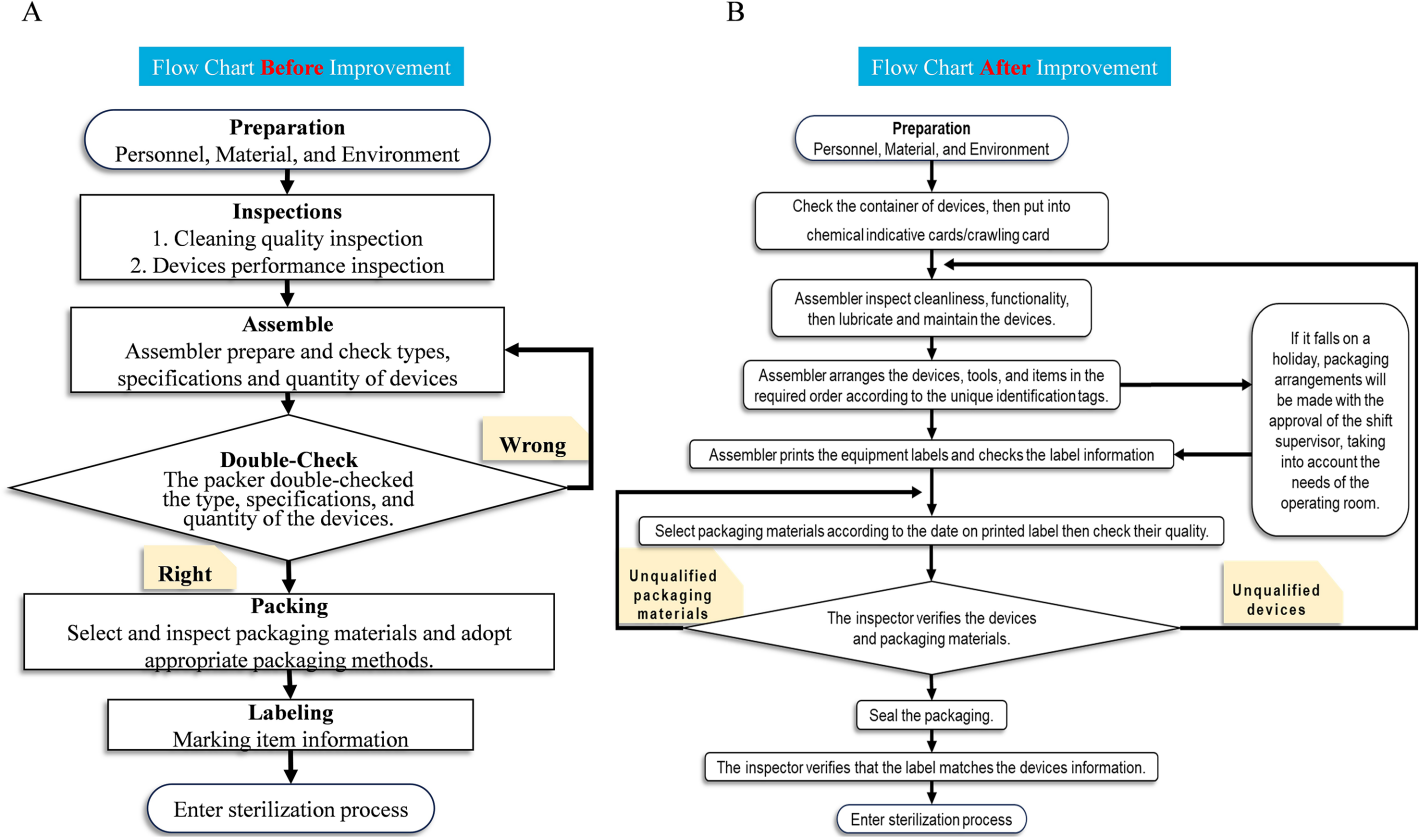
Workflow optimization in surgical instrument packaging following QCC intervention. **(A)** Flow chart of the packaging workflow before QCC implementation, showing relatively simple inspection and assembly steps with limited verification controls. **(B)** Flow chart of the optimized workflow after QCC implementation, demonstrating enhanced standardization, additional verification steps, clearer responsibilities, and strengthened process control.

Comparison of packaging workflow charts before and after the QCC intervention showed notable improvement in process standardization, clarity of operational steps, and implementation discipline. The optimized flow structure contributed to reduced procedural variability and strengthened execution of inspection protocols (**Figure 3****).** Continuous quality tracking confirmed that improvement effects were stable rather than transient, with sustained maintenance of the defect rate at the target level during prolonged follow-up.

### Economic and operational benefit outcomes

**Figure 4:**
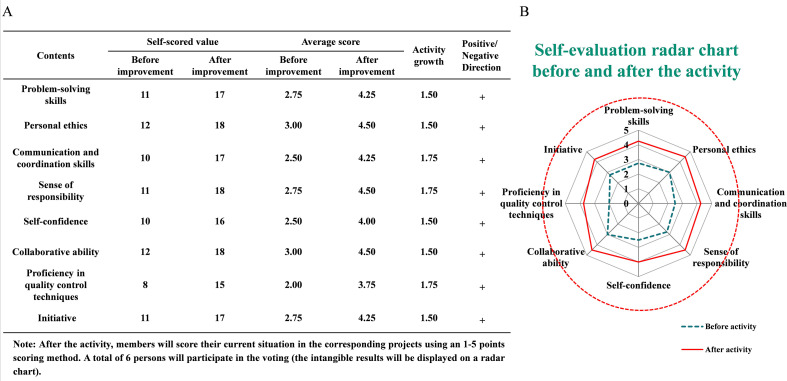
Improvement in comprehensive team capability following QCC implementation. **(A)** Comparison of team self-evaluation scores before and after QCC implementation, showing notable improvements across multiple competency dimensions. **(B)** Radar chart illustrating enhanced problem-solving, communication, responsibility, collaboration, confidence, initiative, and quality control proficiency following QCC activities.

In addition to measurable outcomes, intangible benefits were evident. Radar chart assessment showed improvements in multiple domains including staff professional competence, quality awareness, execution capability, teamwork, and problem-solving initiative (**Figure 4**). In addition to quality improvement outcomes, implementation of the QCC intervention produced measurable and potentially significant economic and operational benefits. Based on an estimated annual workload of approximately 400,000 surgical device packs, and a documented reduction in the non-conforming rate from 0.213% to 0.106% (absolute reduction 0.107%), it is estimated that approximately 428 defective device packs could be avoided annually. Assuming that 15% of unqualified packs result in device damage or loss requiring replacement, and applying an average procurement cost of 2,000 yuan per device, the avoided annual equipment replacement expenditure is calculated as approximately 128,400 yuan. Similarly, reductions in non-conforming packaging directly decrease the need for repeated instrument reprocessing. Using an estimated reprocessing cost of 50 yuan per item, avoidance of 428 reprocessing events would correspond to an additional annual saving of approximately 21,400 yuan. Moreover, although not directly measured in this pilot, quality improvement and strengthened sterile assurance potentially contribute to lowering healthcare-associated risk, including avoidance of postoperative infection events. Conservatively estimating prevention of even one case of surgical site infection (SSI), with an average additional treatment burden of approximately 50,000 yuan, represents an important clinical and economic gain. Collectively, these findings suggest that improving packaging quality not only enhances operational efficiency and safety reliability but also confers meaningful economic benefit to the institution (**Table 3****).**

**Table 3:** Summary of economic and additional operational benefits associated with quality improvement outcomes.

Benefit domain	Basis of calculation	Estimated value
Avoided defective instrument packs	400,000 packs/year × reduction of 0.107%	428 avoided packs/year
Avoided device loss/replacement	428 × 15% damage × 2,000 yuan	≈ 128,400 yuan/year
Avoided reprocessing costs	428 × 50 yuan	≈ 21,400 yuan/year
Potential avoided infection treatment costs	1 avoided SSI × 50,000 yuan	≈ 50,000 yuan/year
Overall economic and operational benefit	Combined avoided direct and indirect costs	Substantial positive impact

Economic benefits include reduced reprocessing, avoidance of surgical delays, efficiency gains.

## Discussion

The findings of this pilot quality improvement study demonstrate that systematic application of the Quality Control Circle (QCC) model in the CSSD was associated with a significant reduction in the rate of non-conforming surgical instrument packaging, achieving the predefined target of a 50% reduction from baseline. Compared with a baseline non-conformance rate of 0.213%, the post-intervention defect rate reached the computed target of 0.106% corresponding to a ~50% relative reduction, as predicted through our improvement model. The effects were not transient but maintained throughout an extended follow-up period (June–October 2024). Notably, the dominant defect categories nonfunctional/incomplete instruments, missing instruments, and incorrect instrument types showed marked decreases, confirming that targeted QCC measures can structurally optimize quality performance in high-risk processing areas. Our baseline non-conformance rate aligns with previously reported rates of packaging defects in hospital CSSD settings, albeit at the higher end of the spectrum. Several studies have quantified sterile packaging quality outcomes, reporting defect rates predominantly in the range of approximately 1.43‰–1.67‰ (0.143%–0.167%) under rigorous quality inspection frameworks, similar to pre-intervention rates in our cohort (Chen et al., 2022; Yao et al., 2025b). Chen et al. reported surgical package defect rates within this range in a large Chinese hospital cohort, underscoring consistency in dominant defect categories such as incomplete packaging, missing items, and specification errors (Chen et al., 2022). Comparable CSSD quality improvement efforts using structured methodologies have also documented substantial defect reductions. A FOCUS-PDCA quality improvement initiative applied in another Chinese CSSD significantly reduced the distribution defect rate from 1.74% to 0.37% (a ~79% relative decrease), reinforcing the potential of standardized improvement frameworks to drive process quality gains (Huang et al., 2023). These findings indicate that structured, data-driven quality interventions whether QCC or PDCA-based are effective in addressing key failure modes in sterile processing. Other global data also supports the notion that instrument processing errors are amenable to systematic quality interventions. A Lean-oriented improvement study at a U.S. hospital reported a substantial decline in instrument processing errors from 3.0% to 1.5%, with particularly large reductions in packaging and assembly errors (Blackmore et al., 2013). This broader quality improvement evidence highlights that error rates and defect profiles may vary by institution and context, but that targeted process control frameworks consistently yield meaningful improvements.

The success of our QCC intervention likely reflects a confluence of mechanisms. First, rigorous standardization of assembly lists and packaging labels reduced variability in set assembly a known risk factor for instrument mismatches and omissions. The central role of instrument list standardization in reducing packaging defects has been emphasized in other CSSD error-focused research (Pan et al., 2024; Yao et al., 2025b). The enhancing verification protocols and double-check mechanisms introduced redundancy into the packaging process, increasing the likelihood of catching errors before final processing. Redundancy and cross-checks have been highlighted in safety literature as key contributors to error reduction in complex workflows like sterile processing (Alfred et al., 2021). The maintenance and inspection enhancements ensured instruments were functional and compliant with procedural standards before packaging, thereby minimizing nonfunctional or defective instrument errors post-processing. Event tracking and equipment maintenance logs, commonly referenced in CSSD quality systems, also support downstream safety and reduced reprocessing needs (Jing et al., 2022). The workflow optimization and competency reinforcement through structured training improved execution fidelity across packaging steps. QCC’s emphasis on frontline engagement and iterative refinement core tenets of continuous improvement methodologies fostered ownership and accountability for quality outcomes, consistent with Total Quality Management (TQM) and Lean principles documented in healthcare quality literature (Yao et al., 2025b).

Although this study did not directly measure clinical outcomes such as surgical site infections (SSI) or other HAIs, improvements in sterile packaging quality are strongly implicated in enhancing perioperative safety. Packaging defects compromise the sterile barrier, increasing contamination risk during storage, transport, and perioperative handling. Decades of surgical safety research emphasize that sterile supply integrity is a critical upstream determinant of operative infection risks, with systematic safety checks (e.g., the WHO Surgical Safety Checklist) demonstrated to reduce perioperative morbidity and mortality in diverse settings (Squires et al., 2025). Defective packaging may also lead to procedural delays, instrument substitutions, or increased handling, all of which correlate with elevated infection risk profiles in observational surgical cohorts. For instance, delayed procedures due to instrument miscounts have been associated with lengthened operative times and subsequent complications a recognized risk factor for SSI. Although quantification of SSI reduction attributable solely to packaging improvements remains an area for future research, the theoretical and empirical linkages to patient safety are compelling and consistent with current quality paradigms.

Beyond safety outcomes, the observed quality enhancement has tangible economic implications. Based on an estimated annual surgical instrument pack workload of ~400,000, achieving a near-50% reduction in defect rates translates to the avoidance of an estimated ~428 defective packs annually. Assuming that a conservative 15% of defective packs result in damaged instruments requiring replacement, and applying an average procurement cost of ¥2,000 per instrument, our QCC intervention could prevent approximately ¥128,400 in replacement expenditures annually. Additional savings from reduced reprocessing costs (~¥21,400 per year) further support the economic value of sustained quality control implementation. Although the direct costs associated with SSIs were not measured in this pilot, a large body of evidence indicates that SSIs impose a substantial economic burden on healthcare systems. Systematic reviews in low- and middle-income settings have shown that the additional healthcare costs attributable to SSIs can range widely, from approximately US $174 to nearly US $29,610 per case, depending on procedure type, setting, and severity of infection, with consistently higher costs compared with non-SSI patients (Monahan et al., 2020). Moreover, narrative and cohort studies across diverse healthcare systems report that each SSI can result in several thousand dollars (or equivalent) of additional direct medical expenditures, extended hospital stays, and increased resource utilization (Piednoir et al., 2021). These cost estimates are supported by observational evidence from China showing that SSIs are associated with prolonged hospitalization and substantial direct economic loss per patient with infected individuals experiencing longer stays and higher associated costs than matched controls (Zuo et al., 2024). Although figures vary by surgical category and institutional context, the range of economic impact underscores that even modest reductions in SSI incidence through upstream quality improvements such as enhanced sterile processing could translate into meaningful cost savings at the institutional and system level.

Taken together, these data suggest that quality interventions targeting sterile processing not only produce measurable operational efficiencies and cost avoidance but also have the potential to contribute to downstream reductions in adverse clinical events that carry significant treatment costs. This study has several strengths. We employed a structured, theory-based quality improvement methodology (QCC with PDCA) that is repeatable and adaptable to diverse CSSD contexts. Our analysis integrated quantitative defect rate monitoring with categorization and process optimization, enabling a comprehensive assessment of both outcome and process changes. Additionally, the sustained improvement over multiple months post-intervention reinforces the durability and clinical relevance of the approach. This was a single-center study without a concurrent control group, which may limit external generalizability. Some comparisons with historical or external benchmarks, while informative, cannot substitute for controlled, multicenter validation. Additionally, we did not capture direct clinical outcomes such as SSI or HAI incidence, precluding causal inference between packaging quality and infection rates. Future studies should pursue larger, multicenter designs that incorporate clinical outcome metrics, economic evaluations, and long-term sustainability assessments. Emerging technologies such as digital tracking, automated instrument recognition, and real-time quality dashboards may further enhance CSSD performance by reducing reliance on manual checks and improving detection sensitivity promising avenues for future research and practice. Integrating electronic performance feedback and artificial intelligence–assisted inspection tools could amplify the gains achieved through human-centric QCC strategies.

## Conclusion

This pilot study demonstrates that structured application of the Quality Control Circle model can substantially improve the quality and safety of surgical instrument packaging in a tertiary hospital CSSD. Through data-driven root cause identification, targeted interventions, and sustained monitoring, the rate of non-conforming packaging was reduced by approximately 50%, with clear optimization of dominant defect categories and stabilization of performance over time. Beyond measurable quality gains, the initiative strengthened process standardization, enhanced professional competency, and fostered a proactive safety culture among CSSD personnel. These findings provide practical evidence that systematic quality management in sterile supply processes is both feasible and impactful, and may contribute meaningfully to reducing downstream risks associated with hospital-acquired infections. The experience offers a replicable improvement model for similar healthcare institutions, while laying a foundation for future multicenter studies to further evaluate clinical outcome benefits and long-term sustainability.

## Data Availability

The raw data supporting the conclusions of this article will be made available by the authors, without undue reservation.
