# Supplementary material for: Application of the quality control circle model to reduce non-conforming surgical instrument packaging and hospital-acquired infection risk: a pilot study
Source: Front Cell Infect Microbiol. 2026 Feb 20;16:1775286. doi: 10.3389/fcimb.2026.1775286 (PMC12963209; doi:10.3389/fcimb.2026.1775286)
Supplement: Supplementary file 1 [file Table1.docx]

**Supplementary Material**

**Title: Application of the Quality Control Circle Model to Reduce Non-Conforming Surgical Instrument Packaging and Hospital-Acquired Infection Risk: A Pilot Study**

**Table S1: Multidisciplinary Composition of the CSSD Quality Control Circle Team and Responsibilities**

| **Date of establishment** | 2023.11.20 | | | |
| --- | --- | --- | --- | --- |
| **Leader** | (Head Nurse，39 years) | | | |
| **Tutor** | (Chief Nurse, Head Nurse，43 years) | | | |
| **Member** | Head Nurse | Undergraduate | 45 years | Drafting plans, formulating countermeasures, reviewing and improving |
|  | Head Nurse | Undergraduate | 43 years | Understanding and analyzing the current situation |
|  | Head Nurse | Undergraduate | 47 years | Implement review |
|  | Disinfection staff | Undergraduate | 37 years | Data statistics and goal setting |
|  | Disinfection staff | Undergraduate | 29 years | Data and image collection, effectiveness confirmation |
| **Department** | Disinfection Supply Center | | | |
| **Schedule** | 2023.11.20-2024.06.10 | | | |


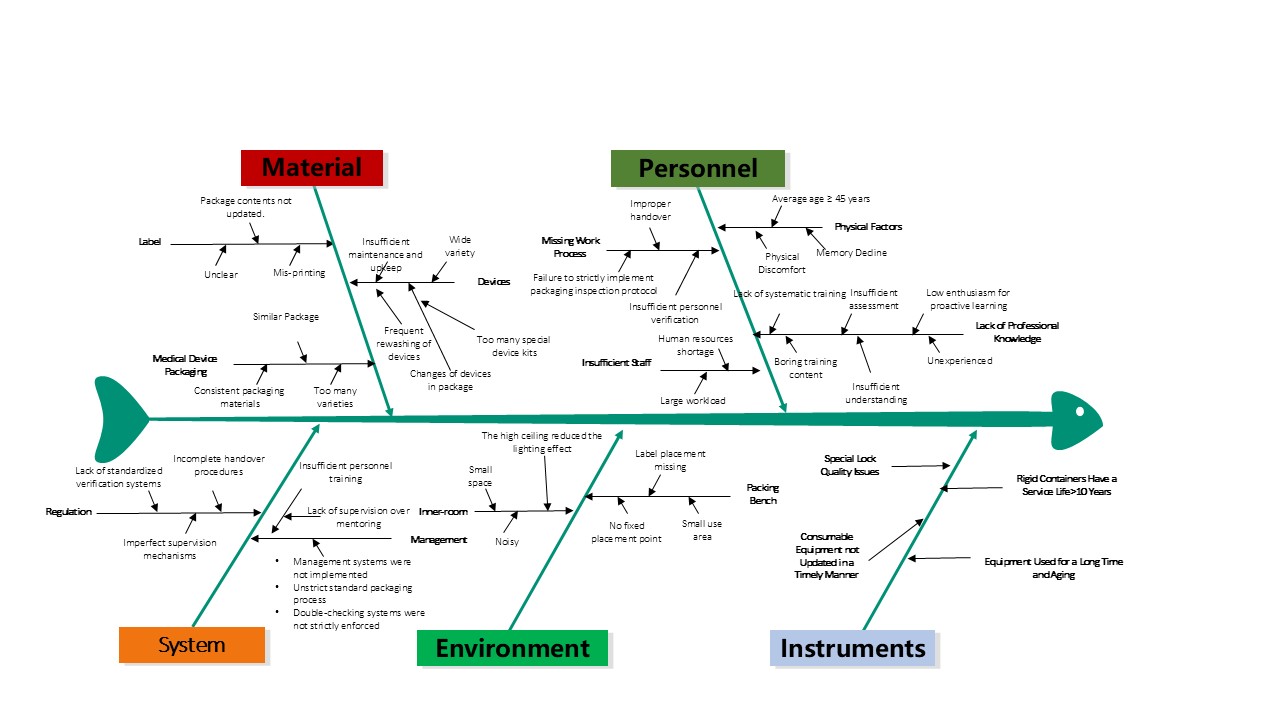


**Supplementary Figure 1. Fishbone (Ishikawa) root cause analysis of non-conforming surgical instrument packaging prior to QCC intervention.**

The fishbone root cause analysis identifying factors contributing to packaging non-conformance in the CSSD. Causes were categorized into six domains—personnel, process, environment, equipment, management, and system—and highlighted key issues including insufficient training, poor adherence to inspection protocols, inadequate supervision, deteriorated identification systems, and inadequate instrument maintenance. These findings guided the design of targeted QCC interventions.

**Table S2. Tangible Outcomes After Rectification (May 1–31, 2024); Total surgical device packs inspected: 19,060 | Total non-conforming: 38 | Defect rate: 0.199%**

| **Inspection cycles** | **Starting time** | **Ending time** | **Packaging defect factors** | | | | | | | |
| --- | --- | --- | --- | --- | --- | --- | --- | --- | --- | --- |
|  |  |  | **Devices inside the sterile pack not fully functional** | **Missing devices in sterile packs** | **Incorrect types of devices inside the sterile pack** | **Devices cleaning not up to standard** | **Wet packs occurred** | **Packaging of sterilized items sealed improperly** | **Substandard chemical indicative cards inside the package** | **Sum** |
| **1** | **May, 1st** | **May, 12th** | 3 | 3 | 2 | 1 | 0 | 1 | 1 | 11 |
| **2** | **May, 13th** | **May, 19th** | 4 | 2 | 2 | 1 | 1 | 1 | 1 | 12 |
| **3** | **May, 20th** | **May, 26th** | 1 | 2 | 1 | 1 | 1 | 0 | 0 | 6 |
| **4** | **May, 27th** | **May, 31th** | 3 | 3 | 0 | 1 | 1 | 1 | 0 | 9 |
| **Sum** | | | 11 | 10 | 5 | 4 | 3 | 3 | 2 | 38 |
| **Percentage** | | | 28.95% | 26.31% | 13.15% | 10.52% | 7.90% | 7.90% | 5.27% |  |
| **Cumulative percentage** | | | 28.95% | 55.26% | 68.41% | 78.93% | 86.83% | 94.73% | 100% |  |
